# Scaling-basis chirplet extracting transform and its application in bearing fault diagnosis

**DOI:** 10.1371/journal.pone.0319497

**Published:** 2025-04-17

**Authors:** Junzhu Zhang, Yating Hou, Liming Wang

**Affiliations:** 1 State Key Laboratory of Dynamic Testing Technology, North University of China, Taiyuan, China; 2 School of Information and Communication Engineering, North University of China, Taiyuan, China; The University of British Columbia, AUSTRALIA

## Abstract

In this paper, we propose a new time-frequency analysis (TFA) method, namely scaling-basis chirplet extracting transform (SBCET). Based on the time-frequency representation (TFR) results obtained by scaling-basis chirplet transform (SBCT), the method introduces a new “extraction operator” to extract the time-frequency (TF) energy associated with the signal to portray the TF energy distribution information of the signal with high accuracy. SBCET can also obtain a TFR with concentrated energy and high resolution for non-stationary signals with close frequency intervals and intense background noise. The effectiveness and superiority are proved by numerical signal processing and experimental verification.

## 1. Introduction

Many natural and synthetic signals are typically non-stationary [1]. Examples include speech, music, and mechanical vibrations. Traditional analysis methods, such as the Fourier Transform (FT) [2], are used to describe the global characteristics of the signal. However, the analysis of non-stationary signals tends to ignore the local information and fails to explain the time-varying characteristics of the signal. TFA addresses this property by transforming a one-dimensional signal into a two-dimensional function, extending the time to a TF property, and describing in detail the signal’s frequency content and its variation with time [3,4]. TFA methods are now widely used in many applications, including the study of seismic signals [5,6], vibration signals [7], biomedical signals [8], and radar signals [9].

Traditional TFA methods such as the short-time Fourier transform (STFT), wavelet transform (WT), and Wigner-Ville distribution (WVD) can diagnose non-stationary signals. However, all of them still have some limitations. For example, the STFT [10,11] cannot achieve high resolution at both low and high frequencies due to the fixed length of the Gaussian window function. WT [12] overcomes the disadvantage of a fixed window size in STFT. However, finding a suitable wavelet basis function is complex, and the method suffers a high degree of redundancy. WVD [13] introduces unwanted additional cross-terms in multi-component signal analysis.

Therefore, a series of parametric time-frequency analysis methods are proposed. For example, the Chirplet Transform (CT) [14] better matches the linearly transformed modulating signal by introducing an additional parameter called chirp rate. However, its window rotation angle is fixed. It cannot achieve ideal results for nonlinear frequency-modulated (FM) and multi-component signals. Several extended versions have been proposed for CT to represent signals with nonlinear FM better. Polynomial CT (PCT) [15] uses a polynomial function instead of the linear FM kernel in the original CT, which can fit the FM law of nonlinear FM signals. Still, the frequency concentration decreases when analyzing multi-component signals, and it is not suitable for analyzing multimode signals. Synchrosqueezing Polynomial Chirplet Transform(SPCT) [16], a synchronous compression PCT for multi-mode amplitude modulation (AM) and FM signals, achieves a highly energy-concentrated TFR by constructing a multikernel operator and an IF estimator. However, it cannot be separated accurately when dealing with signals with similar frequency components. The Generalized Linear Chirplet Transform (GLCT) [17] does not require pre-estimation of parameters and can better handle multi-component signals. However, it produces severe interrupt components when processing signals with small frequency intervals. The velocity synchronous linear chirplet transform (VSLCT) [18] uses a linear chirp basis whose frequency is synchronized with the shaft rotational speed and uses a time-varying window length to respond to changes in signal conditions, improving the time-frequency resolution. Still, the choice of parameters strongly influences it. The scaling-basis chirplet transform (SBCT) [19] extends the CT by scaling the basis at and around the time center in a range of window lengths so that the multi-component signal over the entire window length matches the slope of each instantaneous frequency(IF) trajectory. However, it is subject to high noise interference and some degree of energy dissipation.

In addition, a series of time-frequency post-processing methods have been developed to improve the TF resolution of conventional TFA methods. The spectrogram-based Reassignment Method (RM) [20] redistributes the energy of the signal points to the computed center of gravity position. Recovering the original component from the signal is impossible because it has been redistributed in both the time and frequency domain. Synchrosqueezing Transform (SST) [21,22] based on WT differs from RM because the method compresses and distributes the signal energy only in the frequency direction. However, when analyzing signals with rapidly changing frequencies, SST cannot accurately estimate the true IF of the signal, resulting in signal energy dispersion. Second- and higher-order versions of SST [23,24] have been proposed to address this problem. Still, the Taylor unfolding of the phase comes with a higher time complexity, which increases the computational burden. Multisynchrosqueezing Transform (MSST) [25] based on SST preserves the reconstruction capability of the signal by updating the TF spectrogram to concentrate the ambiguous TF energy through multiple iterations. Synchroextracting Transform (SET) [26] is different from SST theory. SET retains only the TF information related to the IF of the signal and removes most of the surrounding ephemeral energy, which can effectively reduce the influence of noise. A higher-order SET version [27,28] is also proposed to estimate strong frequency modulation signals better.

Although TF post-processing methods have been widely used to analyze non-stationary signals, such TFA methods rely on linear TFA, which is limited by the Heisenberg uncertainty principle. The time resolution and frequency resolution of the linear time-frequency transform cannot be optimized at the same time, so it is required that the constituent components of the multicomponent signal should be sufficiently spaced in the time-frequency plane, i.e., the components have good separability. Therefore, a time-frequency analysis method with good characterization capability must be chosen for multicomponent signals with small frequency intervals. SBCT constructs a new kernel function that allows the chirp rate to vary with frequency and time by scaling the TF basis at and around the corresponding time center. In this way, the corresponding chirplet can precisely match the target slopes of arbitrary window lengths for each trajectory of the multi-component signal, resulting in an accurate TF characterization. However, the frequency resolution of the results is insufficient. It is also susceptible to noise interference. Therefore, inspired by the post-processing method, this paper extends SET to SBCT and proposes a new TFA method called scaling-basis chirplet extracting transform(SBCET). This method outperforms existing TFA methods in analyzing multi-component signals with close frequency intervals and substantial noise interference. It is capable of obtaining TFR results with more concentrated energy.

The paper is structured as follows. Section 2 briefly describes the principles of SBCT and SET and derives the SBCET method principle by formula derivation. Sections 3 and 4 demonstrate the effectiveness of the proposed method by applying it to simulated and experimental signals, respectively, and comparing it with other TFA methods. Conclusions are presented in Section 5.

## 2. Theory

### 2.1 SBCT theory

SBCT is based on CT and is realized by constructing a new phase function. The CT of the signal $x(u)\in L^{2}(R)$ can be expressed as


$$CT(f,t_{c})=\int_{-\infty }^{+\infty }s(u)h(u-t_{c})\exp(-j2\pi \varphi (f,u,t_{c}))du$$ (1)

where s(u) does the Hilbert transform generate the analyzed signal $x\left(u\right)$; $h(u)\in L^{2}(R)$ is a non-negative, symmetric, and normalized real window function, usually a Gaussian function; $\varphi (f,u,t_{c})=fu+C{\left(u-t_{c}\right)}^{2}/2$ is the phase function, $t_{c}\in R$, f∈R denoting time and frequency centers, respectively; C∈R is the chirp rate.

**Fig 1:**
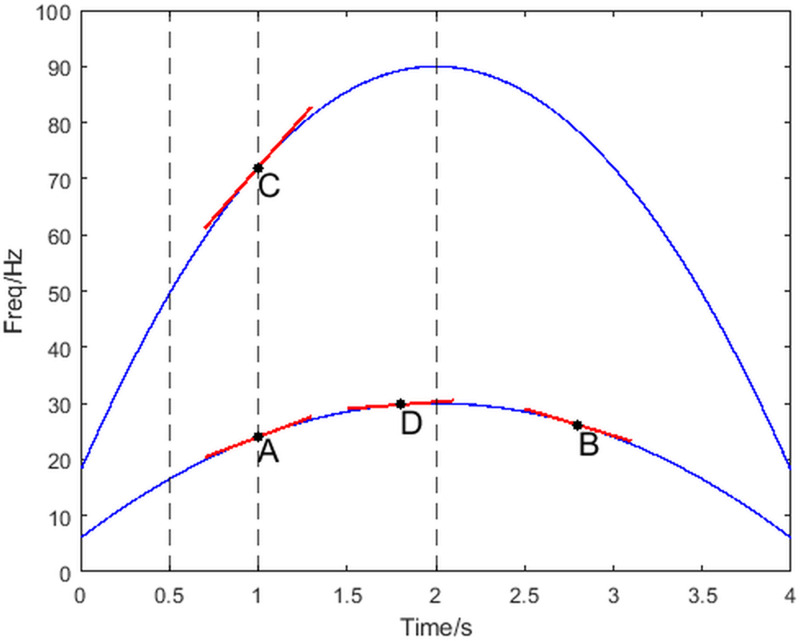
CT limitations.

For multicomponent signals where the IF trajectory varies nonlinearly with time, CT has three limitations. First, the IF trajectory changes over time and requires different chirp rates to match the slopes at different moments (as shown by points A and B in Fig 1). Second, the TF basis cannot match the IF trajectories of all components simultaneously at any given moment (as shown by points A and C in Fig 1). The third limitation is the inability of the TF basis to accurately match the instantaneous frequency trajectories of all targets at each moment of each window length (as shown in Fig 1 at points A and D within 0.5-2s).

To break the three limitations, a new phase function $\varphi_{s}$ is constructed


$$\varphi_{s}(f,u,t_{c},a_{1},a_{2},\ldots ,a_{n})=f\times \left(u+{\sum_{k=1}^{n}a_{k}\left(u-t_{c}\right)}^{1+k}\right)$$ (2)

where $a_{1},a_{2},\ldots ,a_{n}$ the parameter must be determined. The corresponding first and second derivatives can be written as


$$\varphi {'}_{s}=\frac{d\varphi_{s}}{du}=f\times \left(1+\sum_{k=1}^{n}\left(1+k\right)a_{k}{\left(u-t_{c}\right)}^{k}\right)$$ (3)

$$-\tan\left(\theta \right)=\frac{d\varphi {'}_{s}}{du}=f\times \left(\sum_{k=1}^{n}\left(1+k\right)ka_{k}{\left(u-t_{c}\right)}^{k-1}\right)$$ (4)

where $\varphi {'}_{s}$ is the IF trajectory of the phase function $\varphi_{s}$.

Taking the time center $t_{c}$ as a specific window, where *u* obeys $\left(t_{c}-L/2,t_{c}+L/2\right)$, when *u* is equal to the time center $t_{c}$, equation (4) can be written as


$$-\tan\left(\theta \right)=2fa_{1}$$ (5)

where *θ* is the rotating angle of the TF basis at the time center $t_{c}$. Starting from this equation, the value of *θ* varies with the frequency center *f* instead of remaining constant.

Combining the above equations, the SBCT can be expressed as


$$\begin{array}{l} SBCT\left(f,t_{c},a_{1},a_{2},\ldots a_{n}\right)\\ =\int_{-\infty }^{+\infty }s\left(u\right)h\left(u-t_{c}\right)\exp\left(-j2\pi f\times \left(u+\sum_{k=1}^{n}a_{k}{\left(u-t_{c}\right)}^{1+k}\right)\right)du \end{array}$$ (6)

### 2.2 SET theory

SET is a post-processing procedure of the STFT, which retains only the TF information in the STFT results most relevant to the time-varying characteristics of the signal, thus obtaining a time-frequency representation with a higher concentration of energy. The STFT expression of function $s\left(t\right)$ is


$$G\left(t,\omega \right)=\int_{-\infty }^{+\infty }g\left(u-t\right)s\left(u\right)e^{-i\omega u}du$$ (7)

where $g\left(u-t\right)$ denotes a moving window.

We let $g_{\omega }\left(u\right)=g\left(u-t\right)e^{i\omega u}$, equation (7) can be rewritten as


$$\begin{array}{l} G\left(t,\omega \right)={\int_{-\infty }^{+\infty }s\left(u\right)\left(g\left(u-t\right)e^{i\omega u}\right)}^{*}du\\ ={\int_{-\infty }^{+\infty }s\left(u\right)\left(g_{\omega }\left(u\right)\right)}^{*}du=\frac{1}{2\pi }{\int_{-\infty }^{+\infty }\hat{s}\left(\xi \right)\left(\hat{g_{\omega }}\left(\xi \right)\right)}^{*}d\xi \end{array}$$ (8)

Where ${\left(\;\right)}^{*}$ denotes complex conjugation; $\hat{s}\left(\xi \right)$ and ${\hat{g}}_{\omega }\left(\xi \right)$ represent the results obtained after the FT of signal $s\left(u\right)$ and window function $g_{\omega }\left(u\right)$, respectively. $\hat{g_{\omega }}\left(\xi \right)=e^{i\omega t-i\xi t}\hat{g}\left(\omega -\xi \right)$.

Let $G_{e}\left(t,\omega \right)=G\left(t,\omega \right)\cdot e^{i\omega t}$ to get the STFT after the correction


$$G_{e}\left(t,\omega \right)=\frac{1}{2\pi }\int_{-\infty }^{+\infty }\hat{g}\left(\omega -\xi \right)\hat{s}\left(\xi \right)e^{i\xi t}d\xi$$ (9)

The STFT of the signal $s_{h}\left(t\right)=Ae^{i\omega_{0}t}$ gives the following result


$$G_{e}\left(t,\omega \right)=A\cdot \hat{g}\left(\omega -\omega_{0}\right)\cdot e^{i\omega_{0}t}$$ (10)

To calculate the IF estimation factor $\omega_{0}\left(t,\omega \right)$ in the STFT result, it is first necessary to bias the STFT result.


$$\begin{array}{l} \partial_{t}G_{e}\left(t,\omega \right)=\partial_{t}\left(A\cdot \hat{g}\left(\omega -\omega_{0}\right)\cdot e^{i\omega_{0}t}\right)\\ =A\cdot \hat{g}\left(\omega -\omega_{0}\right)\cdot e^{i\omega_{0}t}\cdot i\cdot \omega_{0}=G_{e}\left(t,\omega \right)\cdot i\cdot \omega_{0} \end{array}$$ (11)

From equation (11), one can derive the IF after the short-time Fourier transform1 $\omega_{0}\left(t,\omega \right)$


$$\omega_{0}\left(t,\omega \right)=-i\cdot \frac{\partial_{t}G_{e}\left(t,\omega \right)}{G_{e}\left(t,\omega \right)}$$ (12)

The SET expression is then obtained as follows


$$T_{e}\left(t,\omega \right)=G_{e}\left(t,\omega \right)\cdot \delta \left(\omega -\omega_{0}\left(t,\omega \right)\right)$$ (13)

### 2.3 SBCET theory

The SET algorithm is a post-processing method providing more centralized time-frequency characterization and resolution. However, the results of the time-frequency analysis of this method depend heavily on the quality of the original time-frequency characterization obtained through conventional methods. Therefore, we consider first obtaining the TFR of the signal through SBCT and then estimating the true IF of the signal on that TF domain. In this way, the time-frequency energy distribution information of the signal is portrayed with high precision, and the time-frequency characterization concentration of the signal is improved.

Here, consider the signal $x(t)=e^{\upsilon (t)+j2\pi \omega (t)}$, where ω'(t) is IF. The analytic signal s(u) can be written as


$$s(u)=A(u)\exp\left(j2\pi \omega \left(u\right)\right)$$ (14)

where A(u) denotes the amplitude and ω(u) is the phase function, whose n+1 order Taylor expansion is given by $\omega (u)\approx \omega (t_{c})+\sum_{k=1}^{n+1}\frac{\omega^{\left(k\right)}\left(t_{c}\right)}{k!}{\left(u-t_{c}\right)}^{1+k}$.

The SBCT expression of the signal is


$$SBCT(f,t_{c})=\int_{-\infty }^{+\infty }s(u)h(u-t_{c}){\text{e}}^{-j2\pi \varphi_{s}(f,u,t_{c})}du$$ (15)

Where $\varphi_{s}\left(f,u,t_{c}\right)=f\left(\left(u-t_{c}\right)+\sum_{k=1}^{n}a_{k}{\left(u-t_{c}\right)}^{1+k}\right)$.

For the convenience of subsequent calculations, let $u-t_{c}=\tau$, equations (14) and (15) can be written as


$$s\left(\tau +t_{c}\right)=A\left(\tau +t_{c}\right)\exp\left(j2\pi \left(\omega (t_{c})+\sum_{k=1}^{n+1}\frac{\omega^{\left(k\right)}\left(t_{c}\right)}{k!}\tau^{k}\right)\right)$$ (16)

$$\begin{array}{l} SBCT\left(f,t_{c}\right)=\int_{-\infty }^{+\infty }s(\tau +t_{c})h(\tau )\exp\left(-j2\pi \varphi_{s}(f,\tau +t_{c},t_{c})\right)d\tau \\ =\int_{-\infty }^{+\infty }\begin{array}{l} A\left(\tau +t_{c}\right)\exp\left(j2\pi \left(\varphi (t_{c})+\sum_{k=1}^{n+1}\frac{\varphi^{\left(k\right)}(t_{c})}{k!}\tau^{k}\right)\right)h\left(\tau \right)\\ \cdot \exp\left(-j2\pi \left(f\left(\left(u-t_{c}\right)+{\sum_{k=1}^{n}a_{k}\left(u-t_{c}\right)}^{1+k}\right)\right)\right) \end{array}d\tau \\ =\int_{-\infty }^{+\infty }A\left(\tau +t_{c}\right)h\left(\tau \right)\exp\left(j2\pi \left(\omega (t_{c})-f\tau +\sum_{k=1}^{n+1}\frac{\omega^{\left(k\right)}(t_{c})}{k!}\tau^{k}-f{\sum_{k=1}^{n}a_{k}\tau }^{1+k}\right)\right)d\tau \\ =\int_{-\infty }^{+\infty }A\left(\tau +t_{c}\right)h\left(\tau \right)\exp\left(j2\pi \left(\omega (t_{c})+\left(\omega '(t_{c})-f\right)\tau +\sum_{k=1}^{n+1}\left(\frac{\omega^{\left(k+1\right)}(t_{c})}{\left(k+1\right)!}-fa_{k}\right)\tau^{1+k}\right)\right)d\tau \end{array}$$ (17)

Thus, there is:


$$\begin{array}{l} \left|SBCT(f,t_{c})\right|\\ =\int_{-\infty }^{+\infty }\begin{array}{l} A\left(\tau +t_{c}\right)h\left(\tau \right)\\ \cdot \exp\left(j2\pi \left(\omega (t_{c})+\left(\omega '(t_{c})-f\right)\tau +\sum_{k=1}^{n}\left(\frac{\omega^{\left(k+1\right)}(t_{c})}{\left(k+1\right)!}-fa_{k}\right)\tau^{1+k}\right)\right) \end{array}d\tau \\ \leq \left|\int_{-\infty }^{+\infty }A(\tau +t_{c})h(\tau )d\tau \right| \end{array}$$ (18)

The equation in (18) holds when the following two equations, (19) and (20), are satisfied; and $\left|SBCT(\omega ,t_{c})\right|$ takes the maximum value, i.e., the highest energy concentration is obtained.


$$\omega '\left(t_{c}\right)-f=0\;\;\text{i.e.}\;\;\omega \text{’}\left(t_{c}\right)=f,$$ (19)

$$\frac{\omega^{\left(k+1\right)}(t_{c})}{\left(k+1\right)!}-fa_{k}=0\;\;\text{i.e.}\;\;\frac{\omega^{\left(k+1\right)}(t_{c})}{\omega '(t_{c})\left(k+1\right)!}=a_{k}$$ (20)

Where k=1,2,…n.

Next, we need to find the IF $\omega '(t_{c})$ of the signal corresponding to the moment $t_{c}$, so we first take the derivative of the signal. According to equation (16), the signal $s\left(\tau +t_{c}\right)$ is derived from time $t_{c}$ as:


$$\partial_{t_{c}}s\left(\tau +t_{c}\right)=s\left(\tau +t_{c}\right)\cdot j2\pi \left(\omega '(t_{c})+\sum_{k=1}^{n}\frac{\omega^{\left(k+1\right)}\left(t_{c}\right)}{k!}\tau^{k}\right)$$ (21)

Then, according to equation (17), the SBCT results for time $t_{c}$ are derived as follows


$$\begin{array}{l} \partial_{t_{c}}SBCT\left(f,t_{c}\right)\\ =\int_{-\infty }^{+\infty }s\left(\tau +t_{c}\right)j2\pi \left(\omega '\left(t_{c}\right)+\sum_{k=1}^{n}\frac{\omega^{\left(k+1\right)}\left(t_{c}\right)}{k!}\tau^{k}\right)h\left(\tau \right)\exp\left(-j2\pi \varphi_{s}\left(f,\tau +t_{c},t_{c}\right)\right)d\tau \\ =j2\pi \omega '\left(t_{c}\right)\int_{-\infty }^{+\infty }s\left(\tau +t_{c}\right)\left(1+\sum_{k=1}^{n}\frac{\omega^{\left(k+1\right)}\left(t_{c}\right)}{\omega '\left(t_{c}\right)k!}\tau^{k}\right)h\left(\tau \right)\exp\left(-j2\pi \varphi_{s}\left(f,\tau +t_{c},t_{c}\right)\right)d\tau \\ =j2\pi \omega '\left(t_{c}\right)\int_{-\infty }^{+\infty }s\left(\tau +t_{c}\right)\left(1+\sum_{k=1}^{n}\left(1+k\right)a_{k}\tau^{k}\right)h\left(\tau \right)\exp\left(-j2\pi \varphi_{s}\left(f,\tau +t_{c},t_{c}\right)\right)d\tau \end{array}$$ (22)

So there is the following equation


$$\omega '(t_{c})=\frac{\partial_{t}SBCT(f,t_{c})}{j2\pi (SBCT(f,t_{c})+\sum_{k=1}^{n}(1+k)a_{k}SBCT^{\tau^{k}h}(f,t_{c})}$$ (23)

where $SBCT^{\tau^{k}h}(f,t_{c})$ denotes the SBCT result calculated under the window function $\tau^{k}h\left(t\right)$.

Observing the above equation, we can see that to find the IF $\omega '(t_{c})$, we first need to find $\partial_{t}SBCT(f,t_{c})$. Thus, according to equation (15), the following equation can be derived


$$\begin{array}{l} \partial_{t}SBCT(f,t_{c})\\ =-SBCT^{h^{\prime }}(f,t_{c})-j2\pi \int_{-\infty }^{+\infty }s(u)h(u-t_{c})e^{-j2\pi \varphi_{s}(f,u,t_{c})}\partial_{t}\varphi_{s}(f,u,t_{c})du\\ \;=-SBCT^{h^{\prime }}(f,t_{c})+j2\pi \int_{-\infty }^{+\infty }s(u)h(u-t_{c})e^{-j2\pi \varphi_{s}(f,u,t_{c})}f(1+\sum_{k=1}^{n}(1+k)a_{k}{\left(u-t_{c}\right)}^{k})du\\ =-SBCT^{h^{\prime }}(f,t_{c})+j2\pi f(SBCT(f,t_{c})+\sum_{k=1}^{n}(1+k)a_{k}SBCT^{\tau^{k}h}(f,t_{c})) \end{array}$$ (24)

where $SBCT^{h^{\prime }}(f,t_{c})$ denotes the SBCT result calculated under the window function $h'\left(t\right)$.

Substituting equation (24) into equation (23) and simplifying, the IF $\omega '(t_{c})$ becomes


$$\begin{array}{c} \omega '(t_{c})=\frac{-SBCT^{h^{\prime }}(f,t_{c})+j2\pi f(SBCT(f,t_{c})+\sum_{k=1}^{n}(1+k)a_{k}SBCT^{\tau^{k}h}(f,t_{c}))}{j2\pi (SBCT(f,t_{c})+\sum_{k=1}^{n}(1+k)a_{k}SBCT^{\tau^{k}h}(f,t_{c}))}\\ =f-\frac{SBCT^{h^{\prime }}(f,t_{c})}{j2\pi (SBCT(f,t_{c})+\sum_{k=1}^{n}(1+k)a_{k}SBCT^{\tau^{k}h}(f,t_{c}))} \end{array}$$ (25)

Therefore, here, we define the estimator of the IF of the signal as


$$\omega_{0}(t_{c},f)=f-\frac{SBCT^{h^{\prime }}(f,t_{c})}{j2\pi (SBCT(f,t_{c})+\sum_{k=1}^{n}(1+k)a_{k}SBCT^{\tau^{k_{h}}}(f,t_{c}))}$$ (26)

Using the mathematical delta function, the SBCT TFR of the signal is used to “extract” the TF energy associated with the IF characteristics of the signal, and the SBCET of the signal is defined as


$$SBCET(t_{c},f)=SBCT(t_{c},f)\delta \left(f-\omega_{0}(t_{c},f)\right)$$ (27)

where $\delta \left(t\right)$ is the unit pulse function and $\delta \left(f-\omega_{0}(t_{c},f)\right)$ satisfies the following equation:


$$\delta \left(f-\omega_{0}(t_{c},f)\right)= \{\begin{array}{c} 1,f=\omega_{0}(t_{c},f)\\ 0,f\neq \omega_{0}(t_{c},f) \end{array}$$ (28)

So there is the following expression


$$SBCET(t_{c},f)= \{\begin{array}{l} SBCT(t_{c},f),f=\omega_{0}(t_{c},f)\\ 0,f\neq \omega_{0}(t_{c},f) \end{array}$$ (29)

## 3. Simulation analysis

The SBCET algorithm proposed in this paper can obtain TFR with higher energy concentration for multicomponent signals with close frequency intervals and intense background noise. This section will verify its validity by using single-component signals, frequency-compact, and noise-laden multicomponent simulated signals, respectively, and comparing it with other TFA methods.

### 3.1 Single-component signal analysis

This section explores the concentration of energy in the SBCET proposed in the article. The simulated signals are as follows:


$$x\left(t\right)=\sin\left(2\pi \int_{0}^{t}\left(3\times \left(-6u^{2}+24u+6\right)\right)du\right)$$ (30)

**Fig 2:**
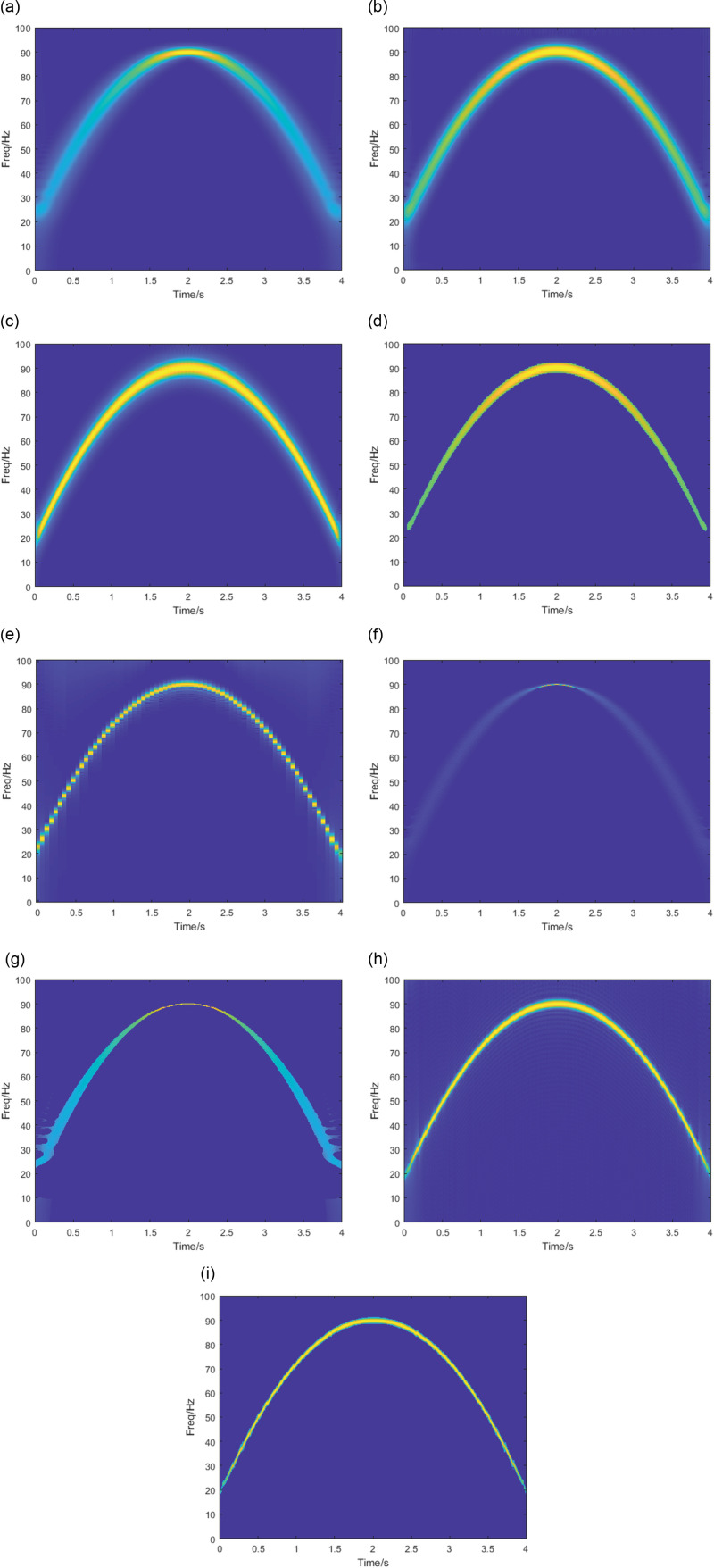
TFA results by (a) STFT, (b)PCT,(c)GLCT,(d)SPCT. (e) VSLCT, (f) SST, (g) SET, (h) SBCT, (i) SBCET.

This section compares STFT, SST, SET, PCT, GLCT, SPCT, VSLCT, and SBCT with SBCET. The window length is set to 128, and the result is shown in Fig 2. It can be seen that the TF plot obtained with the STFT (Fig 2(a)) has the highest energy concentration at the highest point. The energy diverges at the remaining moments. The energy concentration of the PCT and GLCT shown in Figs 2(b) and (c) is improved, but there is still energy dispersed around the signal trajectory (blue-green parts of the figure). The improved SPCT method (Fig 2(d)) based on PCT solves the energy dispersion problem. However, the energy concentration is not high near the peak of the signal. The VSLCT (Fig 2(e)) has a better energy concentration than any of the previous methods. Still, the results present a segmented situation, where it is clear that the overall trajectory of the signal consists of many small segments. The STFT-based post-processing methods SST (Fig 2(f)) and SET (Fig 2(g)) exhibit the same TF distribution as STFT. The results obtained by SBCT (Fig 2(h)) are better than those of the above methods. However, the problem of energy dispersion is still evident, especially in the first and last parts of the signal. SBCET results are shown in Fig 2(i) with smooth trajectories. High energy aggregation is achieved, and the problems of SBCT are solved.

### 3.2 Frequency similarity signal analysis

In this section, the multicomponent signals are constructed as:


$$x\left(t\right)=\sum_{i=1}^{8}\sin\left(2\pi \int_{0}^{t}v_{i}\left(u\right)du\right)$$ (31)

**Fig 3:**
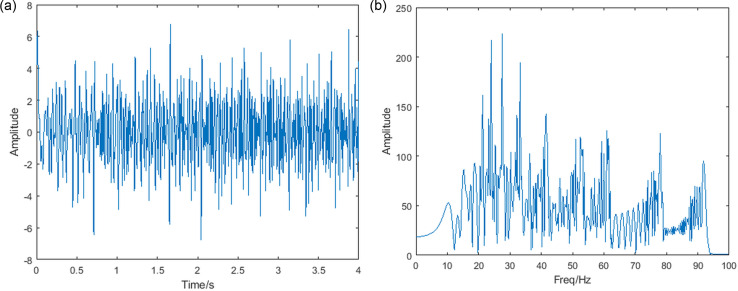
(a) Time domain plot (b) FT image of the simulated signal.

Where, $v_{1}\left(u\right)=-2u^{2}+12u+10$, $v_{2}\left(u\right)$, $v_{3}\left(u\right)$, $v_{4}\left(u\right)$, $v_{5}\left(u\right)$, $v_{6}\left(u\right)$, $v_{7}\left(u\right)$, $v_{8}\left(u\right)$ is the 0.87, 1.2, 1.5, 1.9, 2.2, 2.8, 3.3 times $v_{1}\left(u\right)$. The sampling frequency is 200Hz, and the signal length is 4s. The time domain diagram of the simulated signal is shown in Fig 3(a). The FT of the signal to find its frequency domain result is shown in Fig 3(b). It can be seen that for components whose frequency varies with time, the FT does not capture the change in frequency.

**Fig 4:**
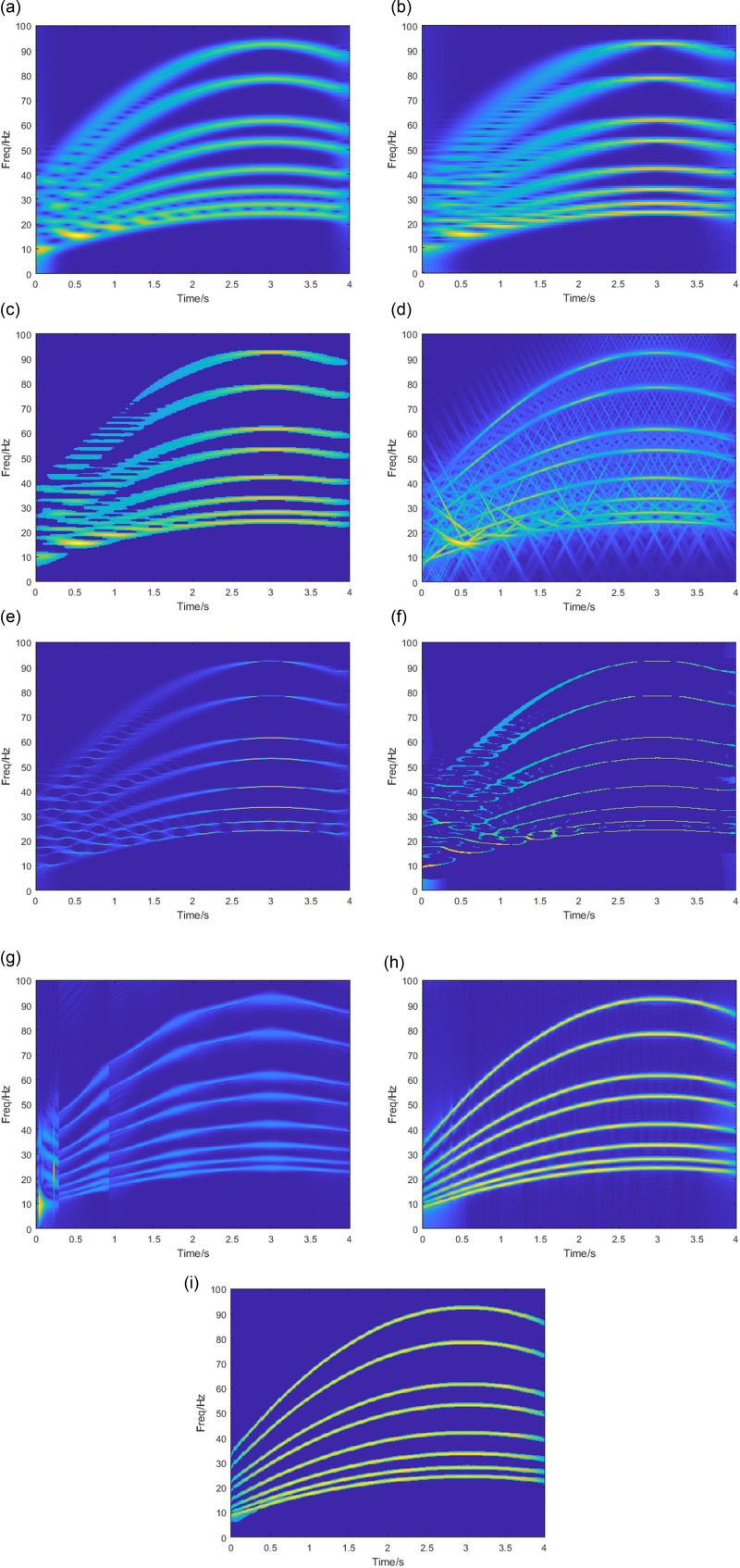
TFA results by (a) STFT, (b)PCT,(c)SPCT,(d)GLCT. (e) SST, (f) SET, (g) VSLCT, (h) SBCT, (i) SBCET.

This section uses the methodology used in section 3.1 for comparisons, with the window length set to 220. The TFA results of each method for the simulated signals are shown in Fig 4. Figs 4(a) and (b) show the results obtained by the STFT and PCT, where the energy is scattered around the IF, forming a wide band and not effectively separated for closely spaced frequency components. The PCT only has a high concentration of energy near 3s. The results obtained from the SPCT (Fig 4(c)) show an increased concentration of energy compared to the PCT. The distribution is consistent with the PCT. The energy concentration of GLCT (Fig 4(d)) is further improved, but it is susceptible to interference. The time-frequency plot shows frequency overlap. It is not possible to separate the close frequency components. Figs 4(e) and (f) show that the SST and SET methods have greatly improved the energy aggregation compared to the STFT method, but they are still unable to separate the components with similar frequencies. The VSLCT method in Fig 4(g) can roughly separate the individual components with closer frequencies, but the energy aliasing phenomenon occurs in 0-0.3s, and the segmentation phenomenon occurs with inconsistent energy concentration in each segment. Fig 4(h) shows that the SBCT method can effectively separate each component near the frequency spacing, but there is some energy divergence in the first and last sections of the signal with lower energy concentration compared to the SBCET method shown in Fig 4(i). In summary, the SBCET method proposed in this paper can separate multicomponent signals with close frequency intervals and achieve high energy concentration.

The Rényi entropy is a generalization of the Shannon entropy and can quantify the stochasticity and uncertainty of the system. In TFA, Rényi entropy is often used as an essential measure of the energy concentration of an algorithm. Lower Rényi entropy indicates that the algorithm has a more energy-focused time-frequency result; thus, a more accurate TFR can be generated. The expression is


$$r=\frac{1}{1-\alpha }\log_{2}\left(\frac{\iint {\left|T(t,\eta )\right|}^{\alpha }dtd\eta }{\iint \left|T(t,\eta )\right|dtd\eta }\right)$$ (32)

where *α* is usually taken as 3 and T(t,η) is the result of the TFA. To quantify the energy concentration of the results of the TFA of each method, the Rényi entropy is given in Table 1, where the SBCET has the smallest value of Rényi entropy. This also demonstrates that SBCET produces TF distributions with the highest concentration of energy compared to other methods.

**Table 1:** Rényi entropy of each TFA method.

TFA	STFT	PCT	SPCT	GLCT	SST	SET	VSLCT	SBCT	SBCET
**Rényi entropy**	16.89	15.22	15.19	17.18	14.67	14.60	15.20	14.93	14.33

**Fig 5:**
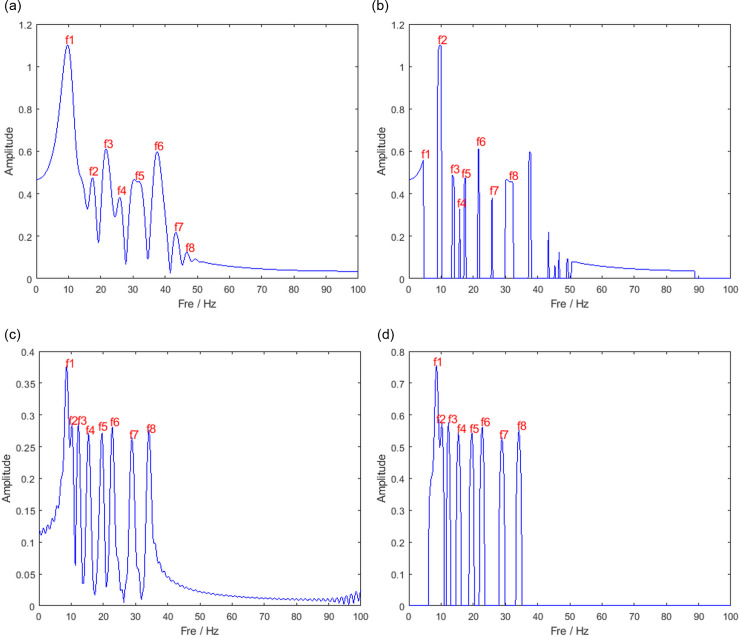
Slices at t = 3s (a) STFT, (b) SET, (c) SBCT, (d) SBCET.

To better observe the aggregation of the signal energy, the time slices of the TF results at t = 3s for the traditional STFT and the extended pre- and post-paper methods (including SET, SBCT, and SBCET) are plotted in Fig 5. As can be seen in the figure, the spacing of the eight frequency components is different in the four methods. The IF estimated by the STFT is somewhat biased due to interference from cross terms, and the signal’s energy is dispersed around the IF. The SET has noise components other than the signal components. The SBCT gives more concentrated TF results, but little energy is dispersed around both ends of the signal, and the amplitude is relatively small at the IF. The method proposed in this paper provides a centralized TFR that accurately describes the amplitude and frequency information of the signal. Therefore, the SBCET offers the best representation of the TF characteristics of the signal.

### 3.3 Noise-added signal analysis

In this section, the effect of noise on the proposed method is explored, and the simulation signals are set up as follows:


$$x\left(t\right)=\sum_{n=1}^{3}\sin\left(k_{n}\int_{t=-\infty }^{t=+\infty }2\pi f\left(t\right)dt\right)+\zeta$$ (33)

**Fig 6:**
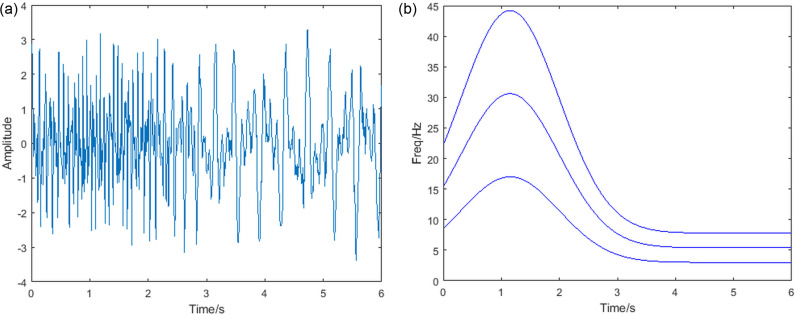
(a) Time-domain plot (b) Theoretical IF trajectory.

Where $k_{1}=1$, $k_{2}=1.8$, $k_{3}=2.6$, $f\left(t\right)=14\exp\left(-0.7{\left(t-1.15\right)}^{2}\right)+3$. *ζ* is the additional Gaussian white noise, set the signal-to-noise ratio(SNR) to 10 dB. Its time-domain plot and theoretical IF trajectory are shown in Fig 6, with a sampling frequency of 100Hz and a signal length of 6s.

**Fig 7:**
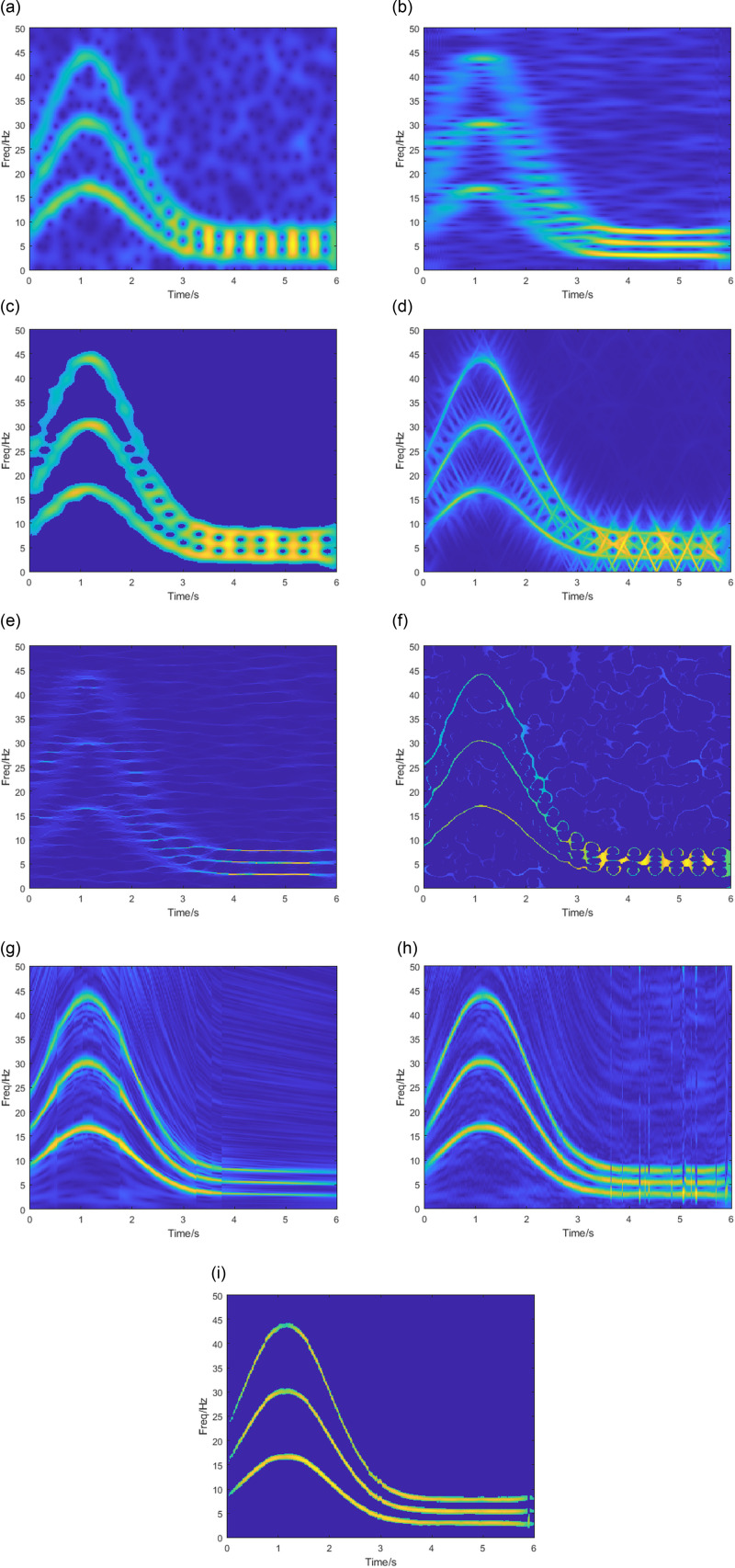
(a) STFT, (b) PCT, (c) SPCT, (d) GLCT, (e) SST, (f) SET, (g) VSLCT, (h) SBCT, (i) SBCET results.

The signals were analyzed using the same methods as in section 3.1, and the results are shown in Fig 7, where the window length is set to 90. Fig 7(a) indicates that the STFT has severe energy divergence at the IF and serious noise pollution within the TF diagram. The results obtained by PCT (Fig 7(b)) are highly affected by noise. It cannot show the signal trajectory accurately. There is energy dispersion in 0-3s and high energy concentration in 3-6s. The results of the time-frequency analysis of the SPCT (Fig 7(c)) are relatively good in terms of noise resistance. However, the energy concentration and time-frequency resolution are not high. The energy overlaps in the part where the frequencies are close. The TF energy concentration of GLCT, SST, and SET in Figs 7(d), (e) and (f) is higher than that of the first three methods, but there is an aliasing phenomenon. The SST is reflected in 0-3.5s. The GLCT and SET are reflected in 3-6s. From Figs 7(g) and (h), it can be seen that the SBCT and VSLCT TF energy is more concentrated compared with the STFT, but all of them still have the phenomenon of energy dispersion and are affected by noise. The SBCET method proposed in this paper is shown in Fig 7(i), it can be seen that compared to other algorithms, SBCET eliminates most of the noise disturbances within the TF diagrams and improves its readability while maintaining a very high accuracy of the IF estimation.

**Fig 8:**
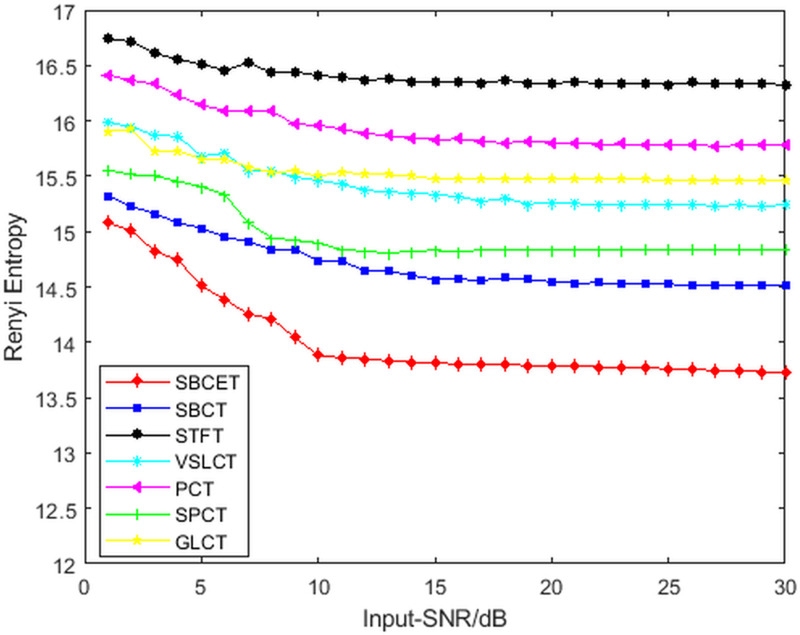
Rényi entropy of TFA methods with different SNR.

To explore the effect of noise on the proposed method, Gaussian white noise with varying degrees of SNR (1-30 dB) was added to the simulated signals. The simulated signal is then processed using each of the above TFA methods. Since the TFR obtained by the SET and SST are insufficient to show the exact frequency trajectory of the signal, a comparison of the Rényi entropy of the results processed by the remaining TFA methods is shown in Fig 8. As can be seen from the figure, as the SNR increases, the corresponding Rényi entropy values all become smaller. This indicates that the energy concentration of its TFR becomes higher, and the Rényi entropy value of the SBCET is always minimized under the same conditions. The results show that the Rényi entropy value calculated using the method proposed in this paper is minimized regardless of the level of noise pollution. This confirms that the method proposed in this paper has higher energy concentration when dealing with noisy signals.

## 4. Experimental validation

**Fig 9:**
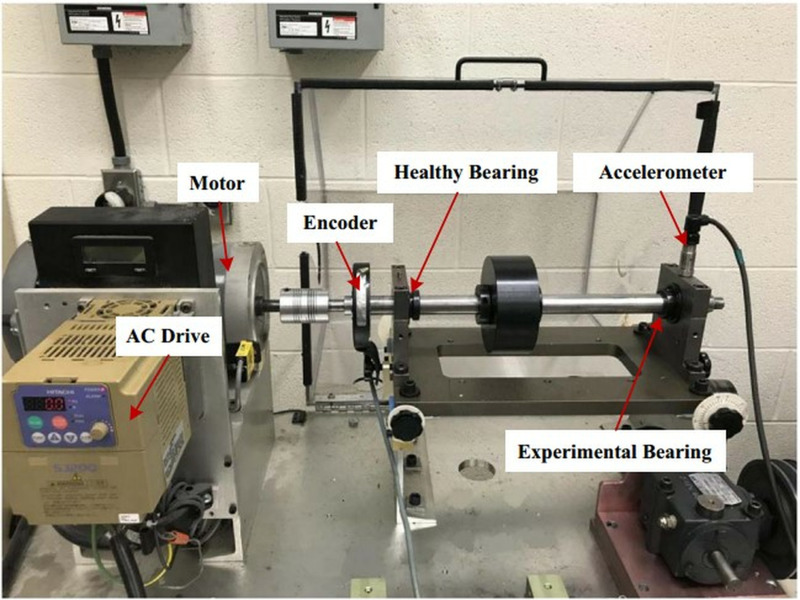
Experimental setup.

As an essential part of rotating machinery, bearings can significantly impact production if they fail during actual operation. The current identification of bearing faults still relies on extracting the characteristic frequency of bearing faults. To verify the effectiveness of the proposed method, rolling bearing vibration signals at time-varying speeds are analyzed. Data from publicly available datasets from the Ottawa University [29]. The experimental setup was the SpectraQuest machinery fault simulator (MFS-PK5M). Vibration data is collected by an ICP accelerometer placed on the housing of the experimental bearing. The specific experimental setup is shown in Fig 9.

The entire unit is driven by an electric motor, and the speed is regulated by an AC drive. Two ER16K ball bearings are located at each end of the shaft. It should be noted that on the left are healthy (normal) bearings, and on the right are replaceable bearings, which usually correspond to faulty bearings. Table 2 clearly shows the structural parameters of the studied rolling bearings. According to the parameters of the bearing, the ball-pass frequency (BPFI) of the inner race of the bearing is equal to the product of the fault characteristic frequency (FCF) coefficient (5.43) and the shaft rotational frequency, i.e., $BPFI=5.43f_{r}$. Similarly, the ball-pass frequency of the outer-race(BPFO) of the bearing $BPFO=3.57f_{r}$. The sampling frequency was set to 200kHz, and the sampling duration was 10s throughout the test.

**Table 2:** Bearing parameters.

Bearing type	Pitch diameter	Ball diameter	Number of balls	BPFI	BPFO
ER16K	38.52 mm	7.94 mm	9	5.43 $f_{r}$	3.57 $f_{r}$

### 4.1 Failure signal analysis of bearing inner ring

**Fig 10:**
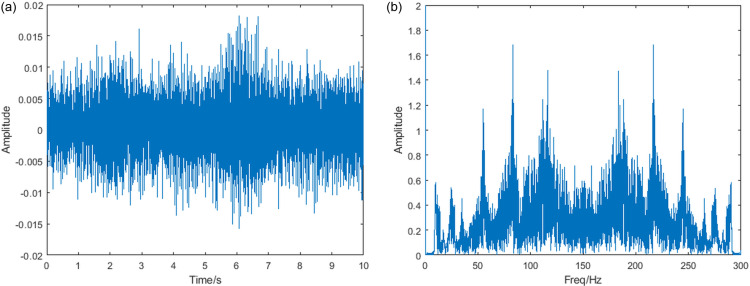
Vibration signal (a) time domain plot (b) FFT result.

To verify the method’s validity, the inner ring failure data I-C-2 is selected for analysis in this section. The rotational speed increases from 14.1 Hz to 23.5 Hz and then decreases to 18.0 Hz. A downsampling operation must be performed since the high sampling rate results in too much data. The sampling rate after downsampling is 5000 Hz. Its time-domain diagram is shown in Fig 10(a). Fig 10(b) shows the result of the fast Fourier transform(FFT) performed on it. From the figure, it can be seen that the fault characteristic frequency component and rotational frequency component cannot be accurately identified at variable speeds.

**Fig 11:**
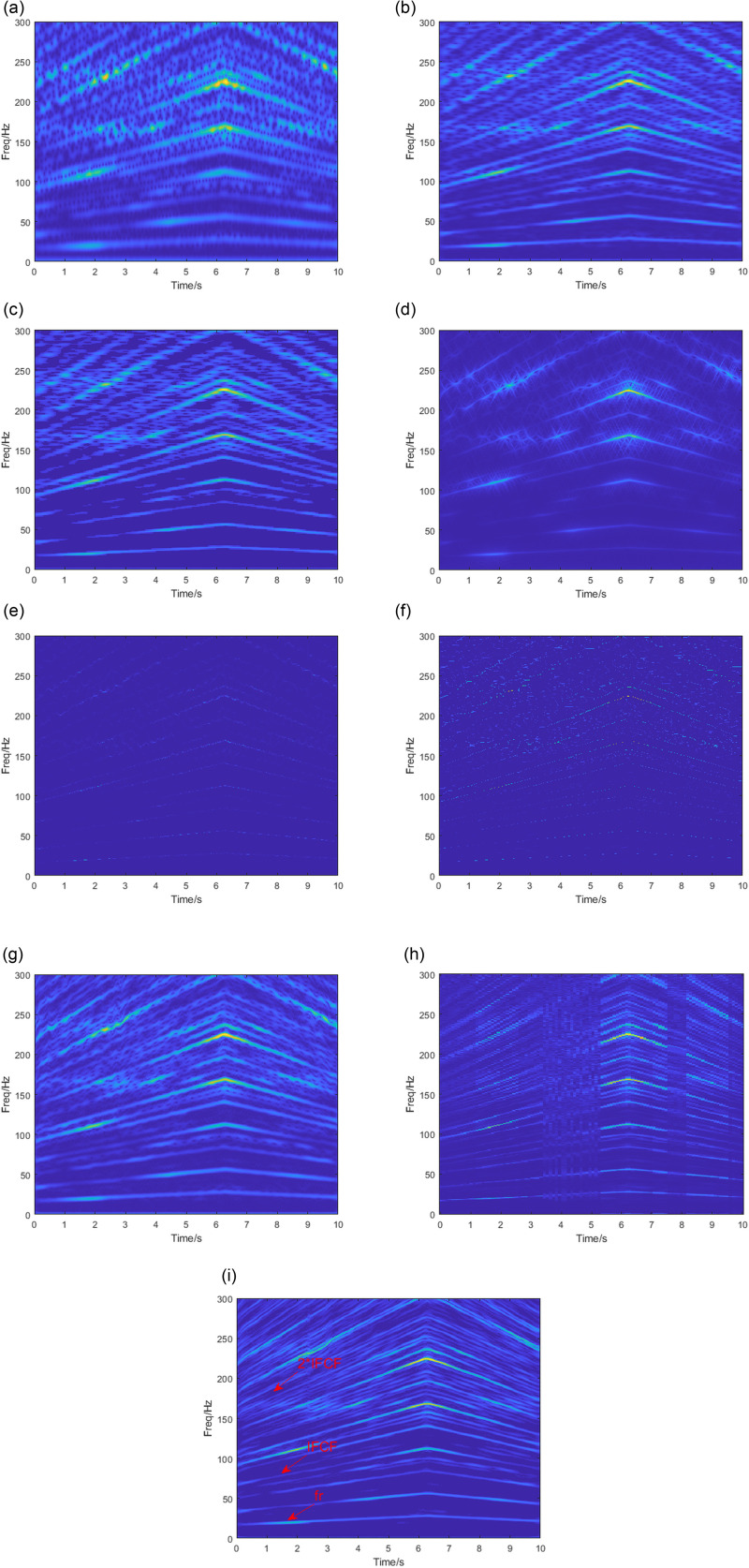
TFA results by (a)STFT, (b) PCT, (c) SPCT, (d) GLCT, (e) SST, (f) SET, (g) SBCT, (h) VSLCT, (i) SBCET.

The TF diagram obtained by processing using the TFA method mentioned in Section 3 is shown in Fig 11. The window length is set to 200 in each of these methods. The two kernel functions in SBCT are set to 50 and 10. The normalization angle in VSLCT is set to 20. The TF diagrams obtained by the STFT, PCT, SPCT, and SBCT methods shown in Figs 11(a), (b), (c) and (g) can roughly show the frequency characteristics of the vibration signals, but there is a lot of interference from noise in the TF plane. It needs to improve its time-frequency resolution to enhance the ability to portray and characterize time-varying signals. The results of the GLCT analysis (Fig 11(d)) improved the energy concentration but caused the absence of some frequency components. It is not possible to derive the failure frequency correctly. From Figs 11(e) and (f), it can be seen that the frequency characteristics of the TF diagrams obtained by the SST and SET are very weak, and cannot form a continuous frequency trajectory. Rolling bearing fault signals are typically accompanied by significant noise, especially vibration noise from mechanical systems. SET and SST algorithms are sensitive to noise. In the presence of high noise levels, they cannot effectively suppress the noise, which results in the fault features being blurred or masked by the noise, affecting the display quality. Therefore, the fault characteristic frequency cannot be identified. The TF diagram obtained by the VSLCT is shown in Fig 11(h), and its TF resolution is improved compared to the previous methods. However, the energy aliasing phenomenon occurs at 3.5-5.5s and 7.5-8.2s, which cannot distinguish the closely spaced frequency components and effectively extract the fault characteristic frequency. The vibration signal is processed using the SBCET proposed in this paper, and the analysis results are shown in Fig 11(i). The time-varying fault characteristic components (FCF, 2 * FCF) and the speed profile($f_{r}$) can be accurately identified.

### 4.2 Failure signal analysis of bearing outer ring

This section selects the outer ring failure data O-B-2 for analysis. The operating speed decreased from 24.7 Hz to 10.2 Hz. The downsampling process is first performed, and the sampling frequency after downsampling is 6000 Hz.

**Fig 12:**
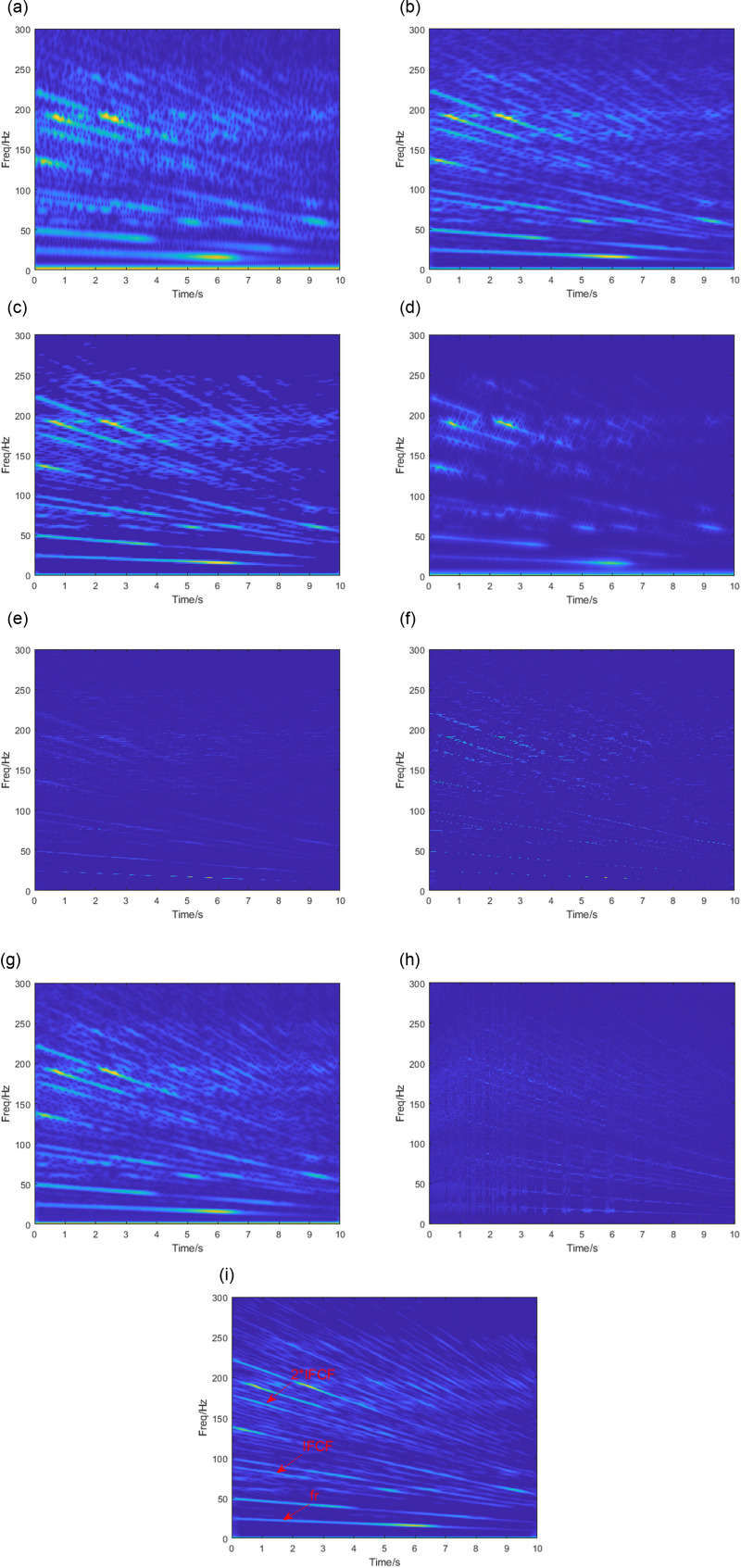
TFA results by (a) STFT, (b) PCT, (c) SPCT, (d) GLCT, (e) SST, (f) SET, (g) SBCT, (h) VSLCT, (i) SBCET.

Fig 12 illustrates the TF results of the vibration signals with the methods used in Section 3. The window length of various TFA methods is set to 200Hz. The two kernel functions in SBCT are set to 40 and 10. The normalization angle in VSLCT is set to 20. The STFT (Fig 12(a)), PCT(Fig 12(b)), and SBCT (Fig 12(g)) can identify most of the fault characteristic frequency components. Still, these two methods are interfered with by noise, and the energy is not concentrated. The SPCT (Fig 12(c)) is minimally disturbed by noise but at the cost of missing some frequency components. The results obtained by GLCT are shown in Fig 12(d). There is energy crossover in the parts with close frequency intervals, and many frequency components are missing. The TF diagrams obtained by the SST, SET, and VSLCT methods are shown in Figs 12(e), (f) and (h), which provide high resolution. Still, they show weakly in the TF diagrams and cannot extract the characteristic frequency effectively. The VSLCT showed severe frequency aliasing in the 0-4s. The analysis results of the SBCET presented in Fig 12(i) portray the fault characteristic frequencies of the vibration signals (including the shaft rotation frequencies $f_{r}$, FCF, and 2 * FCF). In summary, the SBCET method can effectively perform bearing fault diagnosis.

## 5. Conclusion

In this paper, we propose a new TFA method, the SBCET, based on the problems of insufficiently clear identification and poor aggregation in multicomponent non-smooth, nonlinear complex signals that occur in the SET and SBCT. The method firstly obtains the time-frequency spectrum of the signal by SBCT, then estimates the real instantaneous frequency of the signal in the time-frequency domain, uses the frequency immobility point to “extract” the time-frequency energy that is closely related to the time-frequency characteristics of the signal from the original time-frequency spectrum, and removes many fuzzy time-frequency energies, to portray the time-frequency energy distribution information of the signal precisely. Subsequently, simulated signals were used to demonstrate its feasibility. The SBCET can obtain higher TF resolution and noise robustness than other TFA methods. It was successfully applied to rolling bearing fault vibration signals, providing a referenceable technical means for bearing fault detection.
